# Anti-myeloperoxidase antibodies attenuate the monocyte response to LPS and shape macrophage development

**DOI:** 10.1172/jci.insight.87379

**Published:** 2017-01-26

**Authors:** Reena J. Popat, Seran Hakki, Alpesh Thakker, Alice M. Coughlan, Julie Watson, Mark A. Little, Corinne M. Spickett, Paul Lavender, Behdad Afzali, Claudia Kemper, Michael G. Robson

**Affiliations:** 1 Division of Transplant Immunology and Mucosal Biology, MRC Centre for Transplantation, King’s College London, Guy’s Hospital, Great Maze Pond, London, United Kingdom.; 2 School of Life & Health Sciences, Aston University, Aston Triangle, Birmingham, United Kingdom.; 3 Trinity Health Kidney Centre, Department of Clinical Medicine, Trinity College Dublin, St. James’ Hospital Campus, Dublin, Ireland.; 4 MRC and Asthma UK Centre in Allergic Mechanisms of Asthma, King’s College London, Guy’s Hospital, Great Maze Pond, London, United Kingdom.

**Keywords:** Autoimmunity, Inflammation, Monocytes

## Abstract

Anti-neutrophil cytoplasmic antibody (ANCA) vasculitis is characterized by the presence of autoantibodies to myeloperoxidase and proteinase-3, which bind monocytes in addition to neutrophils. While a pathological effect on neutrophils is acknowledged, the impact of ANCA on monocyte function is less well understood. Using IgG from patients we investigated the effect of these autoantibodies on monocytes and found that anti-myeloperoxidase antibodies (MPO-ANCA) reduced both IL-10 and IL-6 secretion in response to LPS. This reduction in IL-10 and IL-6 depended on Fc receptors and enzymatic myeloperoxidase and was accompanied by a significant reduction in TLR-driven signaling pathways. Aligning with changes in TLR signals, oxidized phospholipids, which function as TLR4 antagonists, were increased in monocytes in the presence of MPO-ANCA. We further observed that MPO-ANCA increased monocyte survival and differentiation to macrophages by stimulating CSF-1 production. However, this was independent of myeloperoxidase enzymatic activity and TLR signaling. Macrophages differentiated in the presence of MPO-ANCA secreted more TGF-β and further promoted the development of IL-10– and TGF-β–secreting CD4^+^ T cells. Thus, MPO-ANCA may promote inflammation by reducing the secretion of antiinflammatory IL-10 from monocytes, and MPO-ANCA can alter the development of macrophages and T cells to potentially promote fibrosis.

## Abstract 2

Anti-myeloperoxidase antibodies from patients with anti-neutrophil cytoplasmic antibody (ANCA) vasculitis alter monocyte function in addition to previously described effects on neutrophils.

## Introduction

Anti-neutrophil cytoplasmic antibody (ANCA) vasculitis is a systemic disease with clinical manifestations that include crescentic glomerulonephritis and pulmonary hemorrhage (1). The name ANCA vasculitis reflects the fact that it is characterized by autoantibodies against neutrophils (2). However, these autoantibodies bind to the proteins myeloperoxidase (MPO) (3) or proteinase-3 (PR3) (4), which are found within granules not only in neutrophils but also in monocytes. The evidence that ANCAs are pathogenic comes from in vitro studies in which IgG from patients with anti-MPO or anti-PR3 antibodies activate neutrophils to undergo respiratory burst and degranulation, as first shown by Falk et al. (5) and confirmed by others (reviewed in ref. 6). In vivo support for the pathogenicity of ANCA is provided by studies in which injection of anti-MPO antibodies causes focal necrotizing crescentic glomerulonephritis in mice in a neutrophil-dependent manner (7, 8). However, the effects of ANCA on MPO- and PR3-expressing monocytes have received far less attention.

Monocytes and macrophages are present within early segmental lesions seen on renal biopsies from patients with ANCA vasculitis (9). Therefore, it is important to understand the effects of ANCA on monocytes, as this may contribute to both tissue inflammation and fibrosis. Fibrosis is an important clinical issue in both the lung and kidney, with glomerular sclerosis being closely linked to renal prognosis (10). There are data to suggest functional effects of ANCA on monocytes. Ralston and colleagues described the production of IL-8 in response to PR3-ANCA (11) and O’Brien et al. recently described the production of IL-1β, IL-6, and IL-8 by TNF-α–primed monocytes in response to MPO-ANCA but not to PR3-ANCA (12). Furthermore, production of oxygen radicals by TNF-α–primed monocytes in response to MPO or PR3-ANCA has been described (13). In addition to a pathogenic role for ANCA, cellular immunity is also considered to be important in ANCA vasculitis, with a role for effector CD4^+^ cells demonstrated in a murine model (14). Therefore, the effect of monocytes and macrophages on CD4^+^ T cell activation and how this may be modified by ANCA is also an important question.

Here, we investigated the effect of ANCA on human peripheral blood monocytes and found that MPO-ANCA impacted at least on 2 levels on monocyte function and that these functional deviations are likely to play a significant role in pathogenesis. MPO-ANCA reduced secretion of antiinflammatory IL-10 by monocytes (and may hence further foster local inflammation) and also induced development of macrophages that instruct CD4^+^ T cells, which could contribute to the tissue fibrosis observed in vasculitis.

## Results

### MPO-ANCA decreases IL-10 and IL-6 production from monocytes in response to LPS.

**Figure 1:**
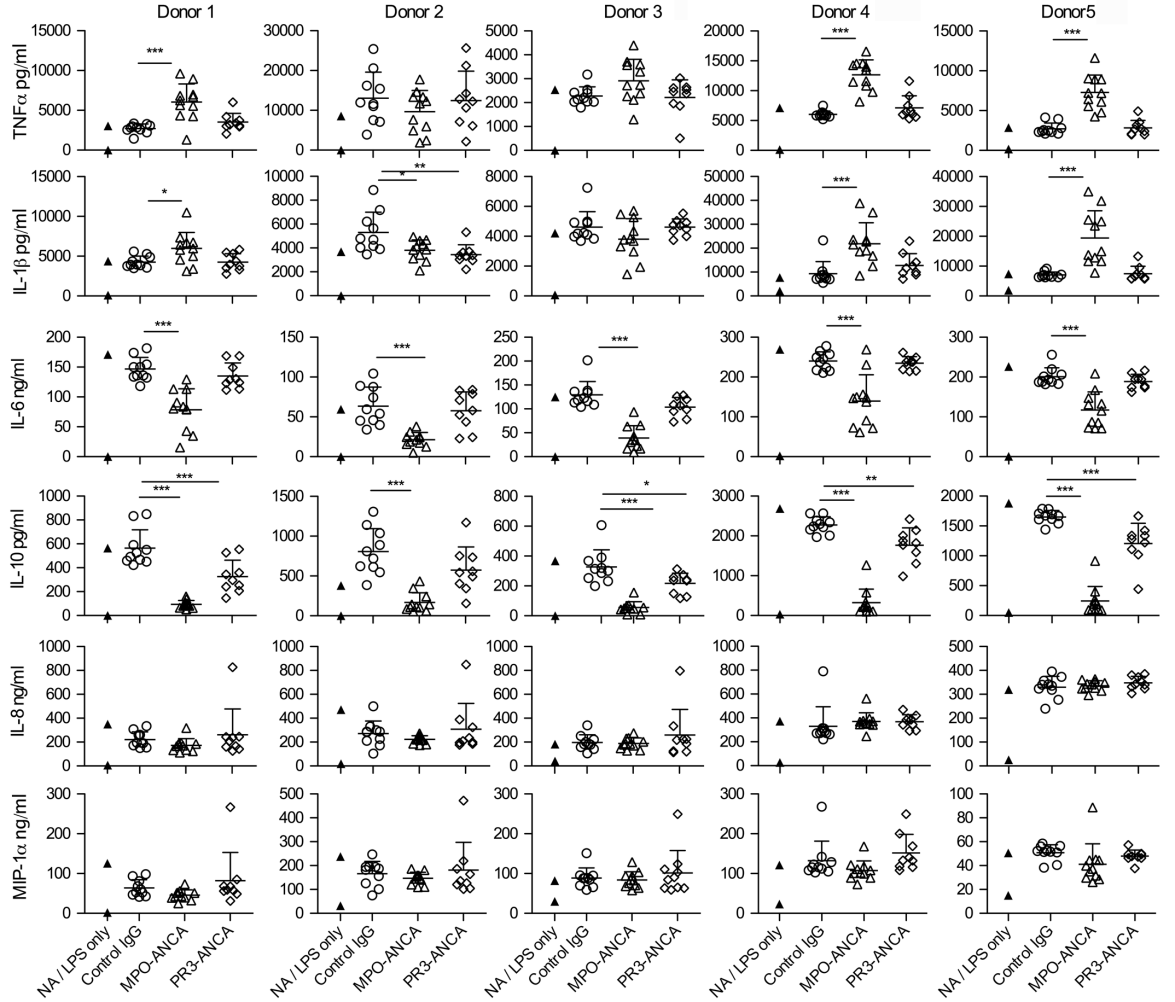
MPO-ANCA decreases IL-10 and IL-6 production from monocytes in response to LPS. Cytokine production from peripheral blood monocytes incubated for 18 hours with 100 ng/ml LPS and anti-myeloperoxidase antibodies (MPO-ANCA), anti–proteinase-3 antibodies (PR3-ANCA), or control IgG (*n* = 9–11 of each type). Experiments were performed in 5 different healthy monocyte donors. Not activated (NA; no LPS) and LPS-only treated cells are shown in the same column, with the LPS-only data being the higher point in all cases. All other groups contained LPS in addition to the stated type of IgG preparation. *P < 0.05, **P < 0.01, ***P < 0.001 by 1-way ANOVA and Dunnett’s post-test with control IgG as the control group. Error bars represent mean ± SEM. For a given monocyte donor, *n* is the number of IgG preparations from different individuals. They are not technical replicates or repeated measures of the same IgG samples.

We examined the effect of a large panel of unselected MPO-ANCA, PR3-ANCA, and control IgG (11, 9, and 10 per group, respectively) on peripheral blood monocytes isolated from 5 healthy donors (see Supplemental Table 1 for patient characteristics; supplemental material available online with this article; doi:10.1172/jci.insight.87379DS1). To mirror the situation where ANCA bind monocytes in the context of an inflammatory response, we stimulated monocytes with LPS and explored whether ANCA modulated the response to LPS. We measured a panel of cytokines in the supernatant of stimulated monocytes stimulated with or without LPS in the presence of control IgG or ANCA for 18 hours. After incubation with MPO-ANCA or control IgG but without LPS, none of the indicated cytokines were detectable (data not shown). With LPS, while we did not observe an effect of MPO-ANCA on IL-8 and MIP-1α production by monocytes, and its effects on TNF-α and IL-1β production were variable among donors, both IL-6 and IL-10 were consistently and significantly decreased by MPO-ANCA across all 5 donors, with the most profound effect seen for IL-10. Addition of PR3-ANCAs only had a consistent downmodulatory effect on IL-10, which was less marked than that of MPO-ANCA (Figure 1). In summary, MPO-ANCA led to a consistent reduction in both IL-6 and IL-10 production by monocytes in response to LPS, while PR3-ANCA only reduced IL-10 but not IL-6 secretion in LPS-primed monocytes. Furthermore, we confirmed that this effect of MPO-ANCA was due to MPO binding activity using affinity-purified antibody (Supplemental Figure 1.)

### The effect of MPO-ANCA on monocytes depends on Fc receptors and enzymatic MPO.

**Figure 2:**
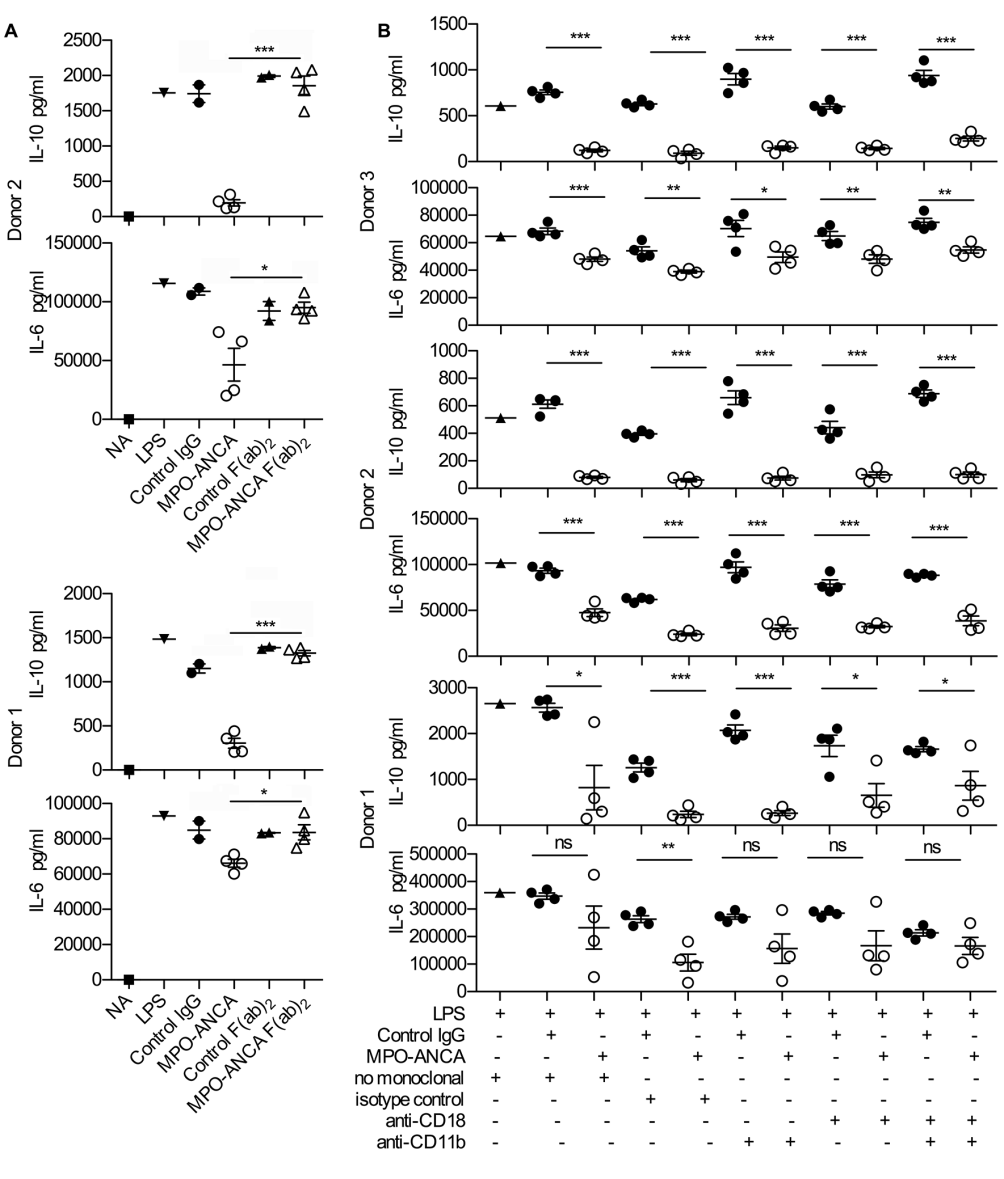
The effect of MPO-ANCA on monocytes depends on Fc receptors but not CD11b or CD18. (**A**) IL-10 and IL-6 production from peripheral blood monocytes incubated for 18 hours with LPS and anti-myeloperoxidase antibodies (MPO-ANCA) (*n* = 4) or control IgG (*n* = 2) compared with the effect of F(ab)_2_ preparations of the same IgG preparations. NA, not activated (no LPS). All other groups contained LPS in addition to the stated type of IgG preparation. (**B**) IL-10 and IL-6 production from peripheral blood monocytes incubated for 18 hours with LPS and MPO-ANCA (*n* = 4) or control IgG (*n* = 4) in the presence of CD11b- or CD18-blocking antibodies. We also tested the effect of combined CD11b and CD18 blockade, with controls shown for no-antibody control and isotype-control monoclonal. ns, not significant. *P < 0.05, **P < 0.01, ***P < 0.001 by 2-tailed Student’s *t* test. Error bars represent mean ± SEM. For a given monocyte donor, *n* is the number of IgG preparations from different individuals. They are not technical replicates or repeated measures of the same IgG samples.

Previously described effects of ANCA on neutrophils have been reported to require Fc receptor binding and involve interaction with the β2 integrin CD18/CD11b, the complement receptor 3 (CR3) (15–17). We therefore examined if this was also the case for the observed effects of MPO-ANCA on monocytes. We generated F(ab)_2_ fragments from 4 MPO-ANCAs and 2 control IgGs and compared responses towards these F(ab)_2_ fragments with whole IgG (Figure 2A) using monocytes from 2 healthy donors. While the MPO-ANCA whole-IgG fraction elicited the expected reduction in IL-6 and IL-10, the addition of MPO-ANCA F(ab)_2_ failed to have any effect on IL-6 or IL-10, strongly indicating that co-engagement of the Fc receptors on the cell surface is also needed for MPO-ANCA effects on monocytes. Interestingly, and in contrast with observations in neutrophils (17), co-engagement of CR3 is not required for MPO-ANCA–induced reduction of IL-6 and IL-10, as blockade of CD18 and/or CD11b did not impact on altered cytokine production (Figure 2B, experiments in 3 monocyte donors).

**Figure 3:**
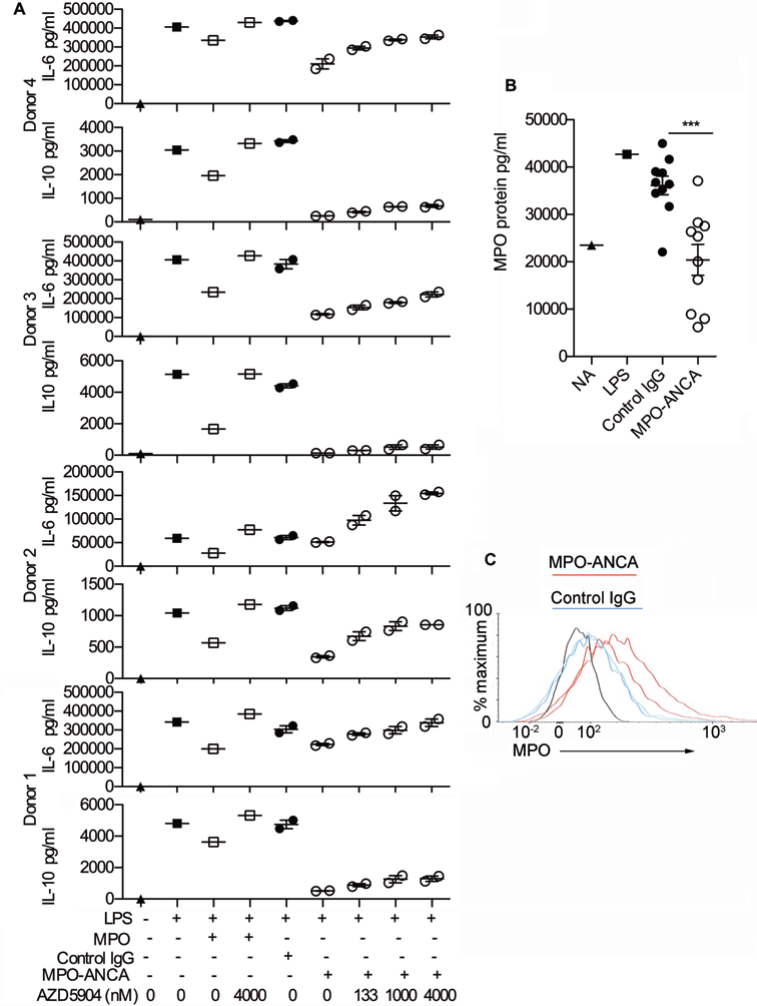
The effect of MPO-ANCA on monocytes depends on enzymatic MPO. (**A**) The effect of varying doses of the myeloperoxidase (MPO) inhibitor AZD5904 on IL-6 and IL-10 production from peripheral blood monocytes incubated for 18 hours with LPS and anti-myeloperoxidase antibodies (MPO-ANCA) (*n* = 2) or control IgG (*n* = 2), with the experiment performed in 4 different monocyte donors. Exogenous MPO (2 μg/ml) with and without AZD5904 (400 nM) was also added to monocytes incubated with LPS. Supplemental Figure 2 shows a dose-response curve for AZD5904 and exogenous MPO. (**B**) MPO measured by ELISA in the supernatant of peripheral blood monocytes incubated for 18 hours with LPS and MPO-ANCA (*n* = 10) or control IgG (*n* = 10). NA, not activated (no LPS). (**C**) Flow cytometry histograms of MPO on peripheral blood monocytes incubated for 2 hours with LPS and MPO-ANCA (*n* = 2, median fluorescence intensities [MFIs] 160 and 206) or control IgG (*n* = 2, MFIs 100 and 99). MPO expression was measured in 2 additional monocyte donors under the same conditions and results were similar. Donor 2: MPO-ANCA (*n* = 2, MFIs 617 and 660) or control IgG (*n* = 2, MFIs 416 and 423). Donor 3: MPO-ANCA (*n* = 2, MFIs 249 and 259) or control IgG (*n* = 2, MFIs 219 and 192). ***P < 0.001 by 2-tailed Student’s *t* test. Error bars represent mean ± SEM. For a given monocyte donor, *n* is the number of IgG preparations from different individuals. They are not technical replicates or repeated measures of the same IgG samples.

Importantly, the MPO-specific portion of MPO-ANCA is equally critical to their monocyte modulatory effects, as is the presence of enzymatically active MPO on monocytes. When we incubated monocytes (± LPS) with exogenously added MPO protein, MPO-ANCA, or control IgG, in the presence or absence of the specific MPO inhibitor AZD5904 (18), we found that both MPO-ANCA and exogenous MPO caused a reduction in IL-6 and IL-10 that is reversed by AZD5904 in a dose-dependent manner (Figure 3A and Supplemental Figure 2). The effects varied between the 4 monocyte donors tested. Overall, the reduction in IL-10 and IL-6 was not as marked for exogenous MPO as for MPO-ANCA, and the effect of AZD5904 was stronger for exogenous MPO. These differences could potentially reflect differing local concentrations of MPO at the monocyte cell membrane with MPO-ANCA and exogenous MPO, respectively. These data suggest that MPO-ANCA is inhibiting IL-6 and IL-10 release in response to LPS by a mechanism dependant on the increased enzymatic activity of MPO. To assess our hypothesis that MPO-ANCA may tether MPO released by activated monocytes to the cell surface, we assessed the surface expression of MPO by flow cytometry in the presence of MPO-ANCA or control IgG and also measured the concentration of MPO in the supernatant of cultured activated monocytes. Addition of MPO-ANCA indeed led to a decrease in free MPO in the supernatant (Figure 3B), with a concomitant increase in cell surface MPO expression (Figure 3C). This increase in cell surface MPO expression did not reflect increased monocyte activation as, in the same experiments, expression of CD11b and CD14 was not higher in MPO-ANCA compared with control IgG–treated samples (data not shown). We also detected immune complexes containing MPO and IgG in the supernatants of monocytes cultured with MPO-ANCA, which further supported our hypothesis (Supplemental Figure 3). In summary, these data suggest that MPO-ANCA may anchor MPO to the surface of the monocyte via Fc receptor binding and thereby facilitate or increase the action of MPO enzyme on the cell surface.

### MPO-ANCA inhibits TLR signaling pathways.

**Figure 4:**
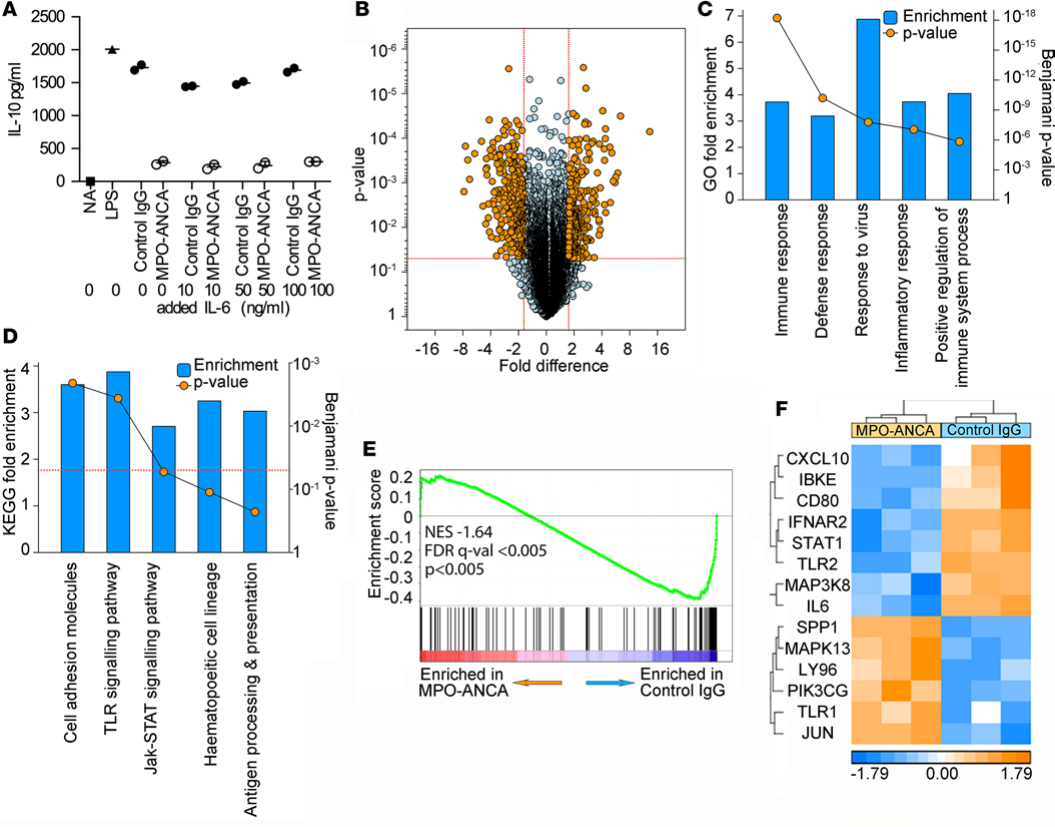
MPO-ANCA inhibits TLR signaling pathways. (**A**) The effect of exogenous IL-6 on IL-10 secretion from peripheral blood monocytes incubated for 18 hours with LPS and anti-myeloperoxidase antibodies (MPO-ANCA) (*n* = 2) or control IgG (*n* = 2). NA, not activated (no LPS). All other groups contained LPS in addition to the stated type of IgG preparation. (**B**) Volcano plot comparing monocytes activated for 6 hours with either MPO-ANCA (*n* = 3) or control IgG (*n* = 3) in the presence of LPS. Threshold for significance is 1.75-fold change in either direction and *P* < 0.05. The 566 differentially expressed transcripts are highlighted in orange (listed in Supplemental Table 2). (**C** and **D**) Gene Ontology (**C**) and KEGG pathway (**D**) functional annotation analysis of the differentially expressed genes, showing the top 6 terms for each. (**E**) Gene set enrichment analysis (GSEA) for enrichment of genes in the KEGG TLR signaling pathway between MPO-ANCA–treated and control IgG–treated monocytes. (**F**) Heat map of the core-enriched genes from the GSEA in **E**. NES, normalized enrichment score; FDR, false discovery rate. In panel **A**, *n* is the number of IgG preparations from different individuals. They are not technical replicates or repeated measures of the same IgG samples.

IL-6 has been shown to promote IL-10 secretion in T cells (19, 20) and we therefore assessed whether the reduction in IL-10 production by monocytes upon ANCA-MPO addition was secondary to the reduction in IL-6. However, in monocytes IL-6 seems not to drive IL-10 production, as addition of exogenous IL-6 to cultures during activation failed to increase IL-10 production (Figure 4A).

To define the pathways driven by MPO-ANCA leading to altered cytokine production in monocytes, we performed gene expression microarrays on monocytes treated with LPS and MPO-ANCA (*n* = 3) or control IgG (*n* = 3) for 1 or 6 hours. At 1 hour, we observed only 12 genes that were differentially expressed between the groups, with a threshold of 1.75 in either direction. However, at 6 hours this rose to 566 genes (Figure 4B, with genes listed in Supplemental Table 2). Functional annotation analysis revealed that the altered gene expression profile was enriched overall for biological processes involved in immune cell activation and inflammatory responses (Figure 4C). Likewise, these analyses demonstrated that one of the most prominently affected KEGG pathways is in genes regulated by TLR signaling (Figure 4D). A gene set enrichment analysis (GSEA) was carried out (21, 22), comparing expression of all genes expressed by MPO-ANCA–treated and control IgG–treated monocytes in our arrays for enrichment of genes in the KEGG TLR signaling pathway, and we found that these genes were preferentially inhibited in the MPO-ANCA–treated cells (Figure 4E). The leading edge or core genes inhibited in this gene set are shown in Figure 4F. Specific examination of the expression of genes known to be regulated by LPS or by the Myd88 pathways using ingenuity pathway analysis (IPA) confirmed a profound inhibitory effect on these genes engendered by the presence of MPO-ANCA relative to control IgG. Z scores for LPS and Myd88 were –4.96 and –2.22, respectively, and overlap *P* values for LPS and Myd88 were 1.87 × 10^–58^ and 1.56 × 10^–9^, respectively (see also Supplemental Figures 4 and 5). Therefore, MPO-ANCA caused a general reduction in normal LPS/TLR–triggered TLR-mediated pathways regulating monocyte activation, which as a net result affected at minimum the production of IL-6 and IL-10 by these cells.

### MPO-ANCA generates oxidized phospholipids.

**Figure 5:**
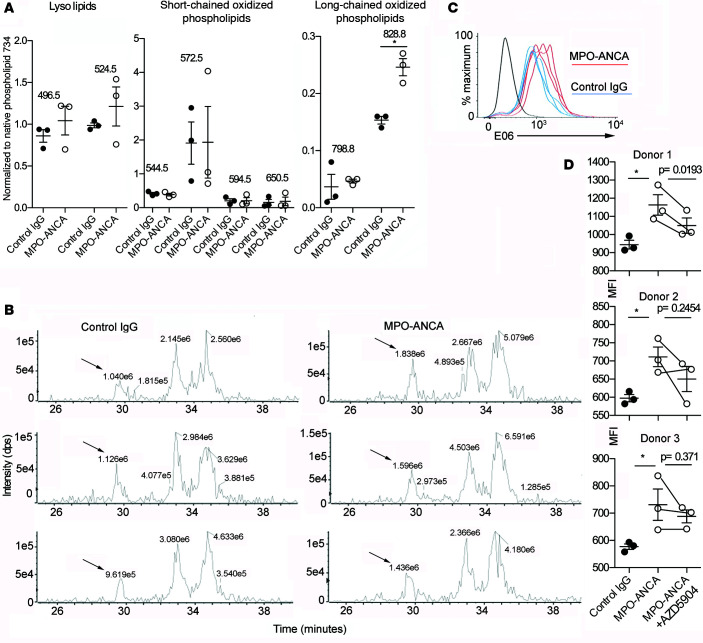
MPO-ANCA generates oxidized phospholipids. (**A**) The most abundant oxidized phospholipids extracted from peripheral blood monocytes incubated for 18 hours with LPS and anti-myeloperoxidase antibodies (MPO-ANCA) (*n* = 3) or control IgG (*n* = 3). The data are presented as a percentage of the intensity of the native saturated phospholipid dipalmitoylphosphatidylcholine at *m*/*z* 734 in each sample. *P < 0.05 by 2-tailed Student’s *t* test). (**B**) Extracted ion chromatograms (XICs) for the control IgG–treated (*n* = 3) and MPO-ANCA–treated (*n* = 3) samples, showing the intensity of the long-chain oxidized phospholipid species at *m*/*z* 828.8 eluting at ~29.5 minutes, ahead of nonoxidized species of the same *m*/*z* ratio. The labels above the peaks indicate their respective area as calculated by Peakview software. Based on these characteristics, the early eluting species at *m*/*z* 828.8 was identified (arrow) as 1-palmitoyl-2-(epoxyisoprostane E2)-*sn*-glycero-3-phosphocholine (PEIPC). The XICs were prepared with a window of 0.7 Da and without smoothing. (**C**) Surface E06 staining on peripheral blood monocytes incubated for 18 hours with LPS and MPO-ANCA (*n* = 3) or control IgG (*n* = 3). (**D**) Median fluorescence intensity (MFI) data from the experiment shown in **C** and 2 other monocyte donors to give 3 monocyte donors in total. The effect of adding the MPO inhibitor AZD5904 (4 μM) to MPO-ANCA–treated monocytes is also shown. Control IgG was compared with MPO-ANCA using a 2-tailed Student’s *t* test. MPO-ANCA was compared with MPO-ANCA + AZD5904 using a 2-tailed paired Student’s *t* test (with lines indicating paired samples). *P < 0.05. Error bars represent mean ± SEM. For a given monocyte donor, *n* is the number of IgG preparations from different individuals. They are not technical replicates or repeated measures of the same IgG samples.

Since MPO is a powerful oxidant that can oxidize phospholipids to form a variety of products (23, 24), and under certain conditions oxidized phospholipids are antagonists of LPS-induced TLR4 activation (25–27), we hypothesized that MPO-ANCA might bind released MPO at the plasma membrane and lead to the generation of oxidized phospholipids. These may be responsible for blocking the effect of LPS. To support this hypothesis, we investigated whether incubation with MPO-ANCA altered the phospholipid profile of LPS-stimulated monocytes and led to increased generation of oxidized phospholipids. We incubated monocytes with LPS and MPO-ANCA (*n* = 3) or control IgG (*n* = 3) for 18 hours, extracted phospholipids, and assessed the phosphatidylcholine (PC) profile using liquid chromatography/mass spectrometry (LCMS). Treatment with MPO-ANCA did not affect the native phospholipid profile, and a variety of chain-shortened oxidized phospholipids were observed in both control IgG– and MPO-ANCA–incubated monocytes (Supplemental Figure 6). Figure 5A shows the relative intensities of 8 oxidatively modified and lyso-PC species that were found to be most abundant. Supplemental Table 3 gives assignments of the PCs based on their mass-to-charge ratio (*m*/*z*) and retention time. The data are presented as a percentage of the signal of the saturated phospholipid dipalmitoyl PC at *m*/*z* 734, as this is relatively resistant to oxidative damage. This approach allows correction for variability in the total phospholipid content of the samples and provides a better assessment of the levels of oxidized species within the total phospholipid pool. For most of the oxidatively modified and lyso-PC species, there was no significant difference in their relative abundance between control IgG–treated and MPO-ANCA–treated monocytes, and the levels of some commonly observed chain-shortened oxidized PCs such as 1-palmitoyl-2-oxovaleroyl-PC (POVPC) and 1-palmitoyl-2-glutaroyl-PC (PGPC) were on the limit of detection. However, it was found that a PC species at *m*/*z* 828.8 eluting in the range of long-chain oxidized phospholipids was significantly higher in MPO-ANCA–treated monocytes (Figure 5A). Figure 5B shows extracted ion chromatograms (XICs) for *m*/*z* 828.8 for each of the 6 samples. This phospholipid was tentatively identified as 1-palmitoyl-2-(5,6-epoxyisoprostane E_2_)-PC (PEIPC). We next investigated the presence of oxidized phospholipids using flow cytometry. We reasoned that this technique was more accessible, allowing us to study multiple donors. Having found some oxidized phospholipids at the limit of detection with MS, it could also be more sensitive, as the oxidized phospholipid is being detected directly on the cell surface. E06 is a monoclonal antibody that binds 1-palmitoyl-2-(5′-oxo-valeroyl)-*sn*-glycero-3-phosphocholine (POVPC) (28). In experiments with 3 monocyte donors, we found that MPO-ANCA significantly increased E06 staining in LPS-primed monocytes (Figure 5, C and D). This inhibition of this effect by AZD5904 was statistically significant in 1 donor with a trend in 2 others (Figure 5D), suggesting a role for enzymatic MPO. Thus, using 2 different techniques, we have found evidence of oxidized phospholipid, which is increased in MPO-ANCA–treated monocytes and can function as a potential TLR4 antagonist, and hence explain the reduced TLR gene expression signature in the presence of MPO-ANCA (Figure 4).

### MPO-ANCA causes an increase in monocyte survival and differentiation to macrophages by increasing CSF-1 expression.

**Figure 6:**
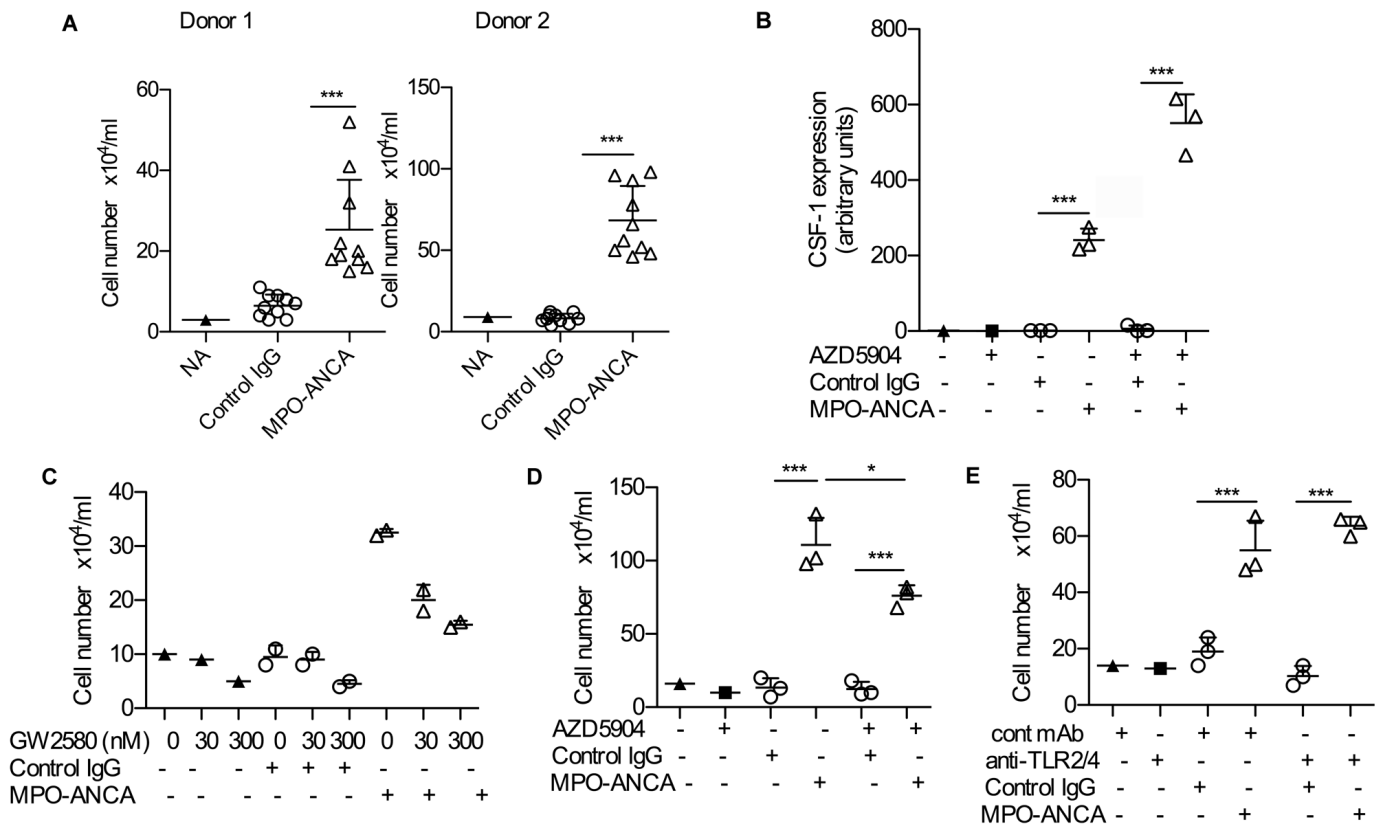
MPO-ANCA causes an increase in monocyte survival and differentiation to macrophages by increasing CSF-1 expression. (**A**) Number of cells remaining after culturing peripheral blood monocytes with 10% human type AB serum and anti-myeloperoxidase antibodies (MPO-ANCA) (*n* = 10) or control IgG for 6 days (*n* = 10) without LPS. Results are shown for 2 healthy monocyte donors. (**B**) CSF-1 gene expression in peripheral blood monocytes after 18 hours in culture MPO-ANCA or control IgG, with and without the MPO inhibitor AZD5904 (400 nM). (**C**) The effect of varying concentrations of the CSF-1 receptor inhibitor GW280 on cell number in the presence of MPO-ANCA (*n* = 2) or control IgG (*n* = 2). (**D**) The effect of the MPO inhibitor AZD5904 (400 nM) on cell number after peripheral blood monocytes were cultured for 6 days in 10% AB serum in the presence of MPO-ANCA (*n* = 3) or control IgG (*n* = 3). (**E**) The effect of TLR2/4 blockade on cell number after 6 days in 10% AB serum in the presence of MPO-ANCA (*n* = 3) or control IgG (*n* = 3). *P < 0.05, ***P < 0.001 by 2-tailed Student’s *t* test. Error bars represent mean ± SEM. For a given monocyte donor, *n* is the number of IgG preparations from different individuals. They are not technical replicates or repeated measures of the same IgG samples.

While monitoring cultures we noticed that monocytes treated with MPO-ANCA were more abundant than those treated with control IgG. Based on these observations, we next explored the effect of MPO-ANCA on monocyte survival and differentiation. To avoid the potential skewing (M1 or M2) effects of growth factor addition, we performed these experiments in the absence of LPS and in the presence of 10% human type AB serum, which has been shown to support monocyte-to-macrophage differentiation in cultures in an unbiased fashion (29). Using 10 MPO-ANCA and 10 control IgG that were also used in the previous experiments, we found that MPO-ANCA significantly increased cell survival after 6 days in culture using monocytes from 2 distinct donors (Figure 6A). Since macrophage colony stimulating factor (M-CSF; CSF-1) is a key survival and differentiation factor for monocytes and macrophages, we measured production of this factor by monocytes. We found indeed a marked increase in *CSF1* mRNA expression in the presence of MPO-ANCA compared with control IgG (Figure 6B). The importance of CSF-1 is supported by the finding that addition of the CSF-1 receptor inhibitor GW2580 caused a dose-dependent reversal of the increase in cell numbers at day 6 (Figure 6C). We next assessed whether the MPO-ANCA/CSF-1–driven effects on monocyte proliferation depended on enzymatic MPO, as we had found this to be the case for the reduction in IL-6 and IL-10. The MPO inhibitor AZD5904 did not prevent the increase in *CSF1* mRNA expression due to MPO-ANCA, which in fact was greater (Figure 6B). Despite the increase in *CSF1* expression, AZD5904 caused a decrease in macrophages at day 6 in the presence of MPO-ANCA (Figure 6D). This suggests that AZD5904 has inhibitory effects on monocyte/macrophage differentiation, unrelated to *CSF1*. Since MPO-ANCA had been shown to interfere with TLR signaling pathways (Figure 4), and human serum contains endogenous ligands for TLR2 and TLR4, we assessed if the observed effect on differentiation and survival was the result of TLR4 antagonism. However, combined blockade of TLR2 and TLR4 with neutralizing Abs had no effect on cell survival (Figure 6E). Overall, these data show a marked increase in cell proliferation after peripheral blood monocytes are incubated for 6 days with MPO-ANCA compared with control IgG. The effect is largely due to an MPO-ANCA–mediated increase in CSF-1 secretion, which itself seems independent of MPO enzymatic activity.

### Macrophages differentiated in the presence of MPO-ANCA express increased CD206, secrete TGF-β, and promote CD4^+^ T cell production of IL-10 and TGF-β.

**Figure 7:**
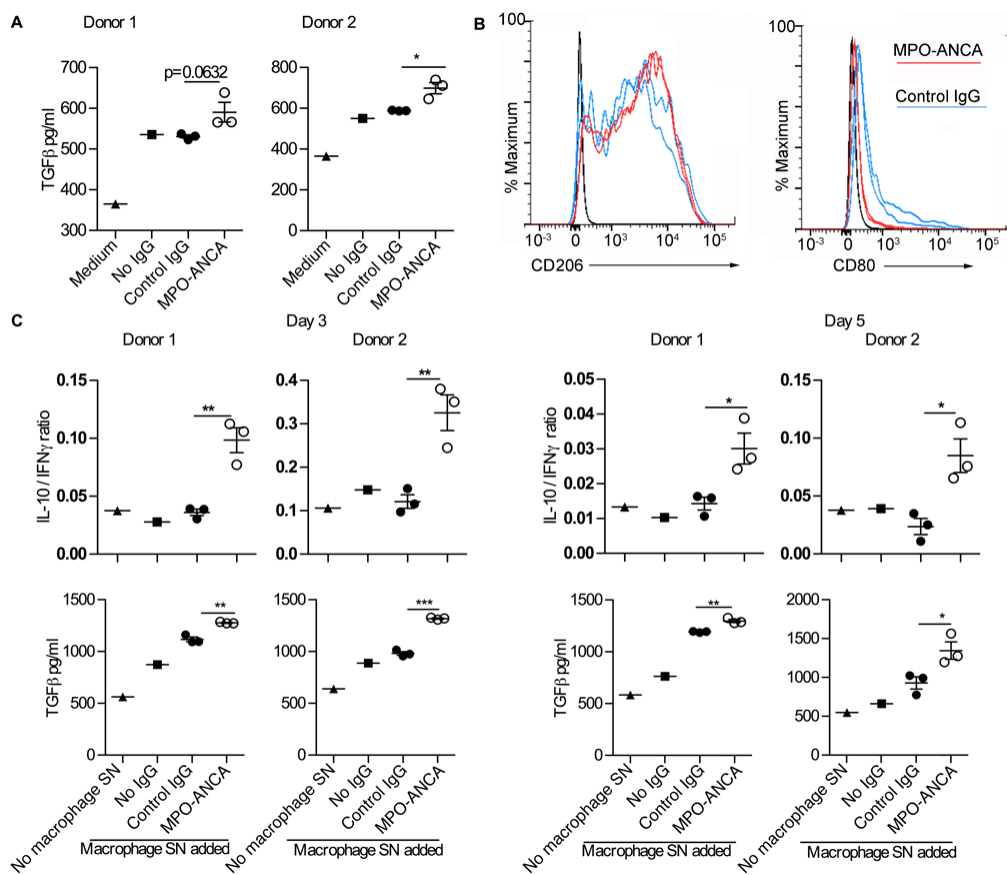
Macrophages differentiated in the presence of MPO-ANCA express increased CD206, secrete TGF-β, and promote CD4^+^ T cell production of IL-10 and TGF-β. (**A**) TGF-β in the supernatants of macrophages that developed from monocytes after 6 days in culture with 10% human type AB serum and anti-myeloperoxidase antibodies (MPO-ANCA) (*n* = 3) or control IgG (*n* = 3). Experiments were performed with monocytes from 2 donors. (**B**) Expression of CD206 and CD80 on macrophages that developed after 6 days in culture with 10% AB serum with control IgG (*n* = 2) or MPO-ANCA (*n* = 2). Experiments were performed with monocytes from 2 healthy donors with histograms shown for 1 donor. CD206 median fluorescence intensities (MFIs) for donor 1 were 3,182 and 3,227 (MPO-ANCA) and 2,202 and 2,624 (Control IgG). CD206 MFIs for donor 2 were 33,466 and 33,287 (MPO-ANCA) and 20,565 and 8,357 (Control IgG). CD80 MFIs for donor 1 were 222 and 228 (MPO-ANCA) and 419 and 465 (Control IgG). CD80 MFIs for donor 2 were 1,113 and 1,213 (MPO-ANCA) and 2,413 and 3,806 (Control IgG). (**C**) Peripheral blood CD4^+^ T cells were stimulated for 3 and 5 days with anti-CD3 and anti-CD28 in the presence or absence of added supernatant from monocyte/macrophages cultured with no added IgG (*n* = 1), control IgG (*n* = 3), or MPO-ANCA (*n* = 3). The ratio of IL-10 to IFN-γ is indicated, with TGF-β concentrations. Concentrations of IL-10, IFN-γ, and IL-17A are shown in Supplemental Figure 8. Experiments were performed with CD4^+^ T cells from 2 donors, each using macrophage supernatants from different experiments. *P < 0.05, **P < 0.01, ***P < 0.001 by 2-tailed Student’s *t* test. Error bars represent mean ± SEM. For a given monocyte donor, *n* is the number of IgG preparations from different individuals. They are not technical replicates or repeated measures of the same IgG samples. SN, supernatant.

To explore the functional consequences of the effect on MPO-ANCA during macrophage maturation in human serum, we measured cytokines in the supernatant at day 6 in culture. IL-10, IFN-γ, IL-17A, IL-2, IL-4, IL-6, and TNF-α were undetectable (data not shown). However, TGF-β was detected at levels above that found in the culture medium, and was specifically increased in the presence of MPO-ANCA (Figure 7A). Furthermore, expression of the macrophage marker CD206 was induced in both MPO-ANCA– and control IgG–treated cultures but was higher with MPO-ANCA (Figure 7B). This difference in CD206 expression was apparent after only 3 days in culture (Supplemental Figure 7). This observation, together with the lower CD80 expression induction we observed in MPO-ANCA–treated monocytes (Figure 7B), suggests that macrophages maturing in the presence of MPO-ANCA have an M2 phenotype in keeping with the role that we have shown for CSF-1 (Figure 6), which is known to favor M2 macrophage skewing (30).

Patients with vasculitis have T cells within affected glomeruli (31) and also changes in and/or persistence of local Th1 and Th2 responses (32). Therefore, to investigate potential effects of MPO-ANCA–exposed macrophages on CD4^+^ T cells, we transferred supernatants from antibody-treated macrophages to CD4^+^ T cells during anti-CD3 and anti-CD28 stimulation and measured cytokine production by stimulated CD4^+^ T cells. While IL-17A was not consistently affected by macrophage-derived factors (Supplemental Figure 8) and IL-4 was not detected (not shown), we observed a consistent decrease in IFN-γ production accompanied with an increase in IL-10 specifically in T cells activated in the presence of supernatants from MPO-ANCA–treated macrophages, with a statistically significant and persistent increase in the IL-10 to IFN-γ ratio (Figure 7C). In addition, TGF-β levels in the T cell supernatants were increased when the T cells were stimulated in the presence of supernatants from MPO-ANCA–treated macrophages (Figure 7C). The TGF-β levels were higher than those found in the macrophage supernatants or the basal T cell medium and so they reflected production from T cells. Overall these data show that in the presence of MPO-ANCA and AB serum, peripheral blood monocytes differentiate into macrophages with an M2-like phenotype that express high levels of CD206, reduced CD80, and secrete increased TGF-β. Furthermore, such MPO-ANCA–conditioned macrophages induce IL-10 and TGF-β production in activated CD4^+^ T cells, which could potentially increase fibrosis.

## Discussion

We have discovered some effects of MPO-ANCA on human monocytes with potential implications for pathogenesis in ANCA vasculitis. These include a marked reduction in both IL-6 and IL-10 in response to TLR4 stimulation in monocytes, which depends on MPO enzymatic activity and is possibly mediated by an MPO-driven increase in oxidized phospholipids. MPO catalyzes oxidation reactions and can produce a variety of oxidized lipids with bioactive effects (33). The PC species at *m*/*z* 828.8 that was increased in MPO-ANCA–treated monocytes was tentatively identified as PEIPC, which has been reported previously to be generated in LPS-stimulated monocytes and neutrophils (34). We also demonstrated an increase in POVPC using the monoclonal antibody E06. Oxidized phospholipids have been shown to have a wide variety of bioactivities, including effects on leukocytes. It is now well established that these effects can be both pro- and antiinflammatory, and depend both on the specific oxidized phospholipid and the cell type (23). One important antiinflammatory mechanism of oxidized PAPC products is through inhibition of TLR2 and TLR4 signaling, as they interfere with LPS and lipopeptide signaling via LBP, sCD14, mCD14, and MD2 (25–27, 35). It has been reported previously that both PEIPC and POVPC, similarly to other oxidized phospholipids, can inhibit LPS-induced E-selectin expression in endothelial cells, at concentrations approximately 10-fold lower than those that induce IL-8 production (27, 36). Interestingly, studies on the role of oxidized phospholipids in immune responses to leprosy have found that PEIPC was able to inhibit human monocyte function in a TLR2-dependent manner (37). PEIPC is indeed one of the most abundant esterified products produced during oxidation of PAPC in vitro, and has also been detected as a major product in inflammatory lesions, as would be found in ANCA vasculitis (24).

Evidence was found for the presence of POVPC on the cell surface by flow cytometry and for free PEIPC in whole-cell extracts, but this does not preclude the occurrence or importance of other oxidized phospholipids or oxidized phospholipid adducts. Currently, it is not clear why a significant increase was only observed for a single oxidized species using MS. If MPO-ANCA tethers MPO to the cell surface, this would cause localized oxidation that might not be readily apparent in extracts of total cellular phospholipids. In addition, some oxidized phospholipids can be interconverted and further metabolized, which may also have contributed to variability in the analysis and limited the number of statistically significant findings. We would also stress that we are not excluding that MPO-ANCA also affects additional pathways that contribute to the observed phenotype.

The effects of IL-6 are difficult to predict. The biology of IL-6 is complex and this cytokine has both proinflammatory and antiinflammatory effects mediated by classical and by *trans*-signaling via a soluble receptor (38). For example IL-6 regulates neutrophil recruitment to inflammatory sites by promoting granulopoiesis (39), but following neutrophil recruitment and soluble IL-6 receptor shedding, IL-6 signaling on stromal cells will limit neutrophil influx (40). In contrast, IL-10 is undeniably an antiinflammatory cytokine and moreover the reduction in its level was more pronounced than that seen for IL-6. Many mechanisms are in place to prevent exaggerated inflammatory responses and IL-10 has proved to be an essential factor in this protection (41). For example, animals that are genetically deficient in IL-10 or are treated with antibodies that neutralize IL-10 die rapidly when infected with pathogens due to the overproduction of proinflammatory cytokines rather than a lack of control of infection (42, 43). In the context of tissue inflammation in ANCA vasculitis, cells such as tissue macrophages, dendritic cells, epithelial cells, and renal mesangial cells do not contain MPO and will not be affected by MPO-ANCA. However, they will be influenced by cytokines released locally by monocytes. A reduction in monocyte IL-10 would therefore be expected to increase the responsiveness of these cells to stimulation by TLRs and other inflammatory stimuli and to lead to an exaggerated inflammatory response. This protective effect of IL-10 is demonstrated in the context of renal inflammation in the unilateral ureteral obstruction model. More severe inflammation develops in the kidney of mice lacking IL-10 compared with wild-type controls (44). Overall, the dominant effect of MPO-ANCA on monocytes is a reduction in IL-10 secretion, which would be expected to promote inflammation and disease in patients.

The TLR4 inhibition observed is specific, as demonstrated by the lack of effect on cytokines other than IL-6 and IL-10. Interesting parallels exist between our work and recent data on monocytes in patients with chronic recurring multifocal osteomyelitis (CRMO) (45, 46). CRMO is an autoinflammatory condition characterized by sterile inflammation in bone with systemic symptoms. In these patients, monocytes fail to produce IL-10 in response to stimulation with LPS (45), whereas production of proinflammatory cytokines including TNF-α and IL-6 are unaffected. Monocytes from CRMO patients had impaired ERK1/2 activation and sp-1 recruitment to the *IL-10* gene promoter (46). The phenotype of CRMO monocytes was reproduced by treatment with an ERK1/2 inhibitor at a concentration having a submaximal effect. At this concentration, IL-10 was reduced with no effect on TNF-α. The molecular mechanism leading to reduced ERK1/2 activation and sp-1 expression was not established in this study. However, this previous work does demonstrate that attenuated TLR4 signaling can lead to a reduction in IL-10, with no effect seen on proinflammatory cytokines.

We also showed that MPO-ANCA causes an increase in survival and differentiation of monocytes to M2-like macrophages in culture primarily through an increase in CSF-1 production. Macrophages play an important role in wound healing as well as fibrosis. They stimulate and activate fibroblasts to produce extracellular matrix proteins (reviewed in ref. 47). It is increasingly recognized that the phenotype of macrophages does not fall into a simple M1 or M2 category (48). However, the increased expression of CD206 and reduced CD80, with TGF-β secretion, suggests that the macrophages fit most closely with an M2-like phenotype, consistent with their induction by an increased concentration of CSF-1 (49). TGF-β has been established for over a decade as a key cytokine that promotes the induction of regulatory T cells (50), and this has been confirmed by subsequent reports. There has been relatively little work on the induction of regulatory T cells by macrophages. In mice, macrophages polarized to secrete TGF-β have been shown to inhibit adriamycin-induced nephrosis by a mechanism that includes the induction of regulatory T cells (51). A further report has also shown that human M2 macrophages can induce CD4^+^ T cells with a regulatory phenotype (52). In both of these examples, the mechanism of suppression was thought to require cell contact, whereas we have shown the effect of a soluble factor in the supernatant. Nonetheless, there are parallels with our data suggesting that M2 macrophages, generated from monocytes incubated with MPO-ANCA, limit T cell activation via TGF-β.

Our current data suggest that macrophages generated from MPO-ANCA–stimulated monocytes act to limit the activation of IFN-γ–producing CD4^+^ T cells and promote the switch to an IL-10–producing phenotype. Over the past few years we have described the life cycle of a Th1 CD4^+^ cell as it changes from producing predominantly IFN-γ to producing both IFN-γ and IL-10 and then to producing predominantly IL-10 and acquiring suppressive capacity, with switching enhanced by ligation of CD46 (53). This represents a key pathway in the life cycle of Th1 cells as they switch from an inflammatory to a regulatory phenotype. These IL-10–secreting CD4^+^ T cells may be similar to, if not the same as, those that have been called induced regulatory T cells (54). We have found that CD46-activated CD4^+^ T cells indeed express increased TGF-β (C Kemper, unpublished data). We now show that CD4^+^ T cells activated in the presence of supernatants from MPO-ANCA–stimulated monocytes/macrophages also produce TGF-β. T cells have been shown to be important in both pulmonary (55) and renal fibrosis (56), with several potential mechanisms that have not been fully defined. A direct effect of TGF-β produced by T cells is one possibility and we have shown how this may be promoted by MPO-ANCA via its actions on monocytes. Effects of TGF-β include a direct stimulation of fibroblasts to cause differentiation into myofibroblasts with concomitant collagen secretion (57). The amount of fibrosis remaining in vital end organs such as the lung and kidney is a major prognostic factor for patients with ANCA vasculitis (10).

Throughout the experiments presented in this paper, we have used IgG from healthy donors as a control. We found that F(ab)_2_ MPO-ANCA failed to have any effect on IL-6 and IL-10 secretion (Figure 2A) and that immune complexes were formed by MPO-ANCA (Supplemental Figure 3). Therefore, an additional informative control would have been to use similarly sized immune complexes with an irrelevant antigen. Further work will be required to assess if at least some of the effects we have described are reproduced by immune complexes containing other antigens.

In summary, we have shown 2 effects of MPO-ANCA on monocytes with implications for disease in patients. These are firstly, a reduction in IL-10 in response to LPS stimulation, potentially due to inhibition of TLR signaling by oxidized phospholipids, which are known to be TLR4 antagonists. We acknowledge that, although our data are consistent with this model, we have not proved that oxidized phospholipids are acting as TLR4 antagonists in this context. Secondly, MPO-ANCA, by stimulating CSF-1 production, promotes monocyte survival and differentiation to macrophages, which instruct the generation of regulatory T cells that produce IL-10 and TGF-β and may promote fibrosis. Further characterization of the phenotype of the human macrophages induced by MPO-ANCA, and in vivo work in murine models, will clarify the potential clinical importance of both of these effects.

## Methods

### Purification of IgG from patients and controls and generation of F(ab)_2_ fragments.

Blood or plasma exchange fluid was obtained from patients with ANCA vasculitis or healthy controls and plasma stored at –80°C. Fibrinogen was precipitated by adding 18 g/100 ml of sodium chloride and IgG was purified with protein G chromatography (GE Healthcare). The endotoxin concentration in the final IgG preparations was measured by Lonza using a LAL kinetic chromogenic assay. For all 30 polyclonal IgGs used, the endotoxin was less than 0.05 eU/250 μg IgG. The concentration of IgG used for all neutrophil and monocyte assays was 250 μg/ml. To generate F(ab)_2_ fragments, whole IgG was buffer exchanged using PD10 columns into 20 mM sodium acetate and digested using immobilized pepsin (Thermo Fisher Scientific). The F(ab)_2_ fragments were separated from undigested IgG using protein A affinity columns (GE Healthcare). SDS-PAGE demonstrated the purity of whole IgG and the F(ab)_2_ fragments. Before all assays, IgG preparations were centrifuged at 16,000 *g* for 30 minutes at 4°C to remove aggregates. In mechanistic experiments where smaller numbers of IgG samples were used, these were taken from this full set of 30 samples.

### Monocyte and CD4^+^ T cell isolation.

Peripheral blood mononuclear cells of healthy human controls were isolated from heparinized whole blood by Ficoll-Paque (GE Healthcare) density gradient centrifugation. Peripheral blood mononuclear cells were aspirated. Monocytes and CD4^+^ T cells were isolated using magnetic CD14 and CD4 microbeads, respectively (Miltenyi Biotec), according to the manufacturer’s instructions.

### Monocyte culture.

Monocytes were resuspended at 1 × 10^6^ cells/ml in RPMI 1640 media containing 10% FBS (Sigma-Aldrich) for 18-hour cultures, or human type AB serum (PAA Labs) for 6-day cultures, and 1% penicillin/streptomycin (Thermo Fisher Scientific). Monocytes were cultured in 48-well plates for selective time periods with or without 100 ng/ml LPS (from *Escherichia coli*, O55:B5, Sigma-Aldrich, L6529) and polyclonal ANCA or control IgG at a final concentration of 250 μg/ml. The cells were incubated at 37°C for varying time points, after which supernatants were collected and cell counts were performed. When used, inhibitors were added prior to the addition of ANCA or control IgG. MPO inhibitor AZD5904 (provided by AstraZeneca) and CSF-1 receptor inhibitor GW2580 (BioVision) were reconstituted in DMSO, with a final DMSO concentration of less than 0.1%. Blocking antibodies were anti-CD11b clone ICRF44 (10 μg/ml), anti-CD18 clone TS1/18 (10 μg/ml) (both Biolegend), and control IgG1 clone 107.3 (BD Biosciences). TLR2/4 blocking and control antibodies were from Invivogen (neutralizing TLR2 Ab clone B4H2, neutralizing TLR4 Ab clone W7C11, and isotype control clone T9C6) and BD Biosciences (isotype control clone 107.3) used at 10 μg/ml each. MPO was detected in monocyte supernatants by ELISA (R&D Systems DuoSet) according to the manufacturer’s instructions. MPO added to the culture in the experiment shown in Figure 5 was from Calbiochem. Recombinant IL-6 was from Biolegend. For macrophage phenotyping, cells were detached using 5 mM EDTA in PBS. Initial culture volumes were 500µl at 10^6^ cells/ml. Cells were counted after detaching with 5mM EDTA in PBS, washing and resuspending in a defined volume of PBS. Reported cell concentrations (Figure 6) are concentrations in a final volume of 300µl (A,C) or 200µl (D,E) respectively.

### Flow cytometry.

Samples were analyzed using a FACSCanto flow cytometer (BD Biosciences) with FACSDiva software (BD Biosciences). At least 10,000 events were collected per sample and data were analyzed using FlowJo software (TreeStar). Antibodies used for flow cytometry were: MPO (clone 5B8, BD Biosciences), CD14 (clone M5E2, BD Biosciences), CD11b (clone D12, BD Biosciences), CD80 (clone 2D10, Biolegend), CD206 (clone 15-2, Biolegend), and TopFluor-E06 (Avanti Polar Lipids). For E06 staining, DAPI was used to exclude dead cells.

### CD4^+^ T cell stimulation.

T cells were activated in 48-well plates coated with antibodies against CD3 (2 μg/ml, clone OKT-3, produced in house from a hybridoma line) and CD28 (2 μg/ml, clone CD28.2, BD Biosciences). The medium was the same composition as the monocyte medium. A final concentration of 10 U/ml recombinant IL-2 was added. Supernatants from the monocyte-to-macrophage differentiated cells were used at a ratio of 2:1 with the T cells. Supernatants were harvested at days 3 and 5 to assay the cytokine profile.

### Cytokine assays and ELISAs.

Th1, Th2, and Th17 cytokines were measured with a Cytokine Bead Array (BD Biosciences) using a FACScalibur (BD Biosciences) and analyzed using CellQuest Pro. IL-1β, IL-8, MIP-1α, CSF-1, and TGF-β levels were measured by ELISA (R&D Systems DuoSet) according to the manufacturer’s instructions.

### Microarray experiments.

RNA was isolated from 5 × 10^5^ monocytes incubated with 100 ng/ml LPS (from *Escherichia coli*, O55:B5, Sigma-Aldrich, L6529) and MPO-ANCA or control IgG (250 μg/ml) for 6 hours using a Qiagen Allprep kit according to the manufacturer’s instructions. Potential genomic DNA contamination was removed by Turbo DNase treatment (Ambion/Life Technologies). Total RNA (5 ng per sample) was amplified and reverse transcribed and labeled using an Ovation Pico WTA System V2 and Encore BiotinIL Kits (NuGen Inc.). The cDNA was hybridized to Illumina HT12V4 microarrays, scanned using an Illumina iScan System, and subjected to quantile normalization within the Genome Studio Suite v1.0 (Illumina). Expression data were analyzed using Genomics Suite (Partek Inc.) version 6.6, IPA (Qiagen), and GSEA (Broad Institute of MIT and Harvard). All original microarray data were deposited in the NCBI’s Gene Expression Omnibus (GEO GSE89153).

### Cellular lipid extraction.

Monocytes from a healthy donor were incubated with anti-MPO or control IgG for 18 hours as described above (3 × 10^6^ cells/condition). The cell pellets were washed and stored at –80°C. All organic solvents used were of HPLC grade. Extraction of phospholipids was performed using a double-extraction technique by addition of 0.5 ml ice-cold methanol containing 0.005% butylated hydroxytoluene and 0.5 ml ultra-high-purity water. The sample was vortexed and 100 μl of a 1 μg/ml solution of 1,2-ditridecanoyl-*sn*-glycero-3-phosphocholine (13:0, 13:0 PC) in methanol containing 0.005% butylated hydroxytoluene was added to each sample. The samples were sonicated at 4°C for 15 minutes. Chloroform (500 μl) was added and the samples were sonicated further as described above. The samples were then centrifuged for 5 minutes at 14,500 *g* and the upper aqueous phase was removed and re-extracted as above. Both organic phases (bottom layer) were combined, dried under a stream of oxygen-free nitrogen, and stored at –80°C. Before analysis, the extracts were reconstituted in 0.2 ml methanol/chloroform (1:1).

### Phospholipid analysis by LCMS.

Phospholipids were separated by reverse phase chromatography on an HLPC system (Dionex Ultimate 3000) controlled by Chromoleon software, using a Proswift RP-4H column (1 mm × 250 mm) at room temperature. The eluents used were (A) aqueous 0.1% formic acid containing 5 mM ammonium formate and (B) methanol containing 0.1% formic acid and 5 mM ammonium formate. The LCMS run time was 50 minutes with a chromatographic gradient of 70% B from 0 to 4 minutes, followed by a 3-step gradient increase to 80% B at 8 minutes, 90% B at 15 minutes, and 100% B at 20 minutes. The gradient was maintained at 100% B until 38 minutes and then decreased back to 70% B by 50 minutes. The flow of the mobile phase was set to 50 μl/minute. Samples were diluted 5-fold or 10-fold in the starting solvent and 10 μl was loaded onto the column. The phospholipids were detected using targeted and data-dependent scanning routines performed on an AB SCIEX 5500 QTrap mass spectrometer controlled by Analyst software. Precursor ion scanning (PIS) of 184 Da was carried out over the mass range 400–1,000 Da to identify PC and oxidized PC. The declustering potential was set to 50 V for all scans and collision energy for PIS was set to 45 eV. Information-dependent data acquisition (IDA) was used to collect MS/MS data based on the following criteria: the most intense ion with +1 charge and minimum intensity of 1,000 cps was chosen for analysis, using dynamic exclusion for 20 seconds after 2 occurrences and a fixed collision energy setting of 47 eV. Other source parameters were adjusted to give optimal response from the direct infusion of a dilute solution of standards.

Data analysis was performed manually using Peakview 2.0 software by generating XICs for individual *m*/*z* ratios corresponding to different modified and unmodified phospholipid species and calculating peak area. The peak area was normalized to that of the internal standard to check for extraction variability, but subsequently the signal intensities were expressed as a percentage of the intensity of the dipalmitoyl PC at *m*/*z* 734 to take into account variability in the total phospholipid present in the samples.

### Reverse transcription quantitative PCR (RT-qPCR).

RNA was isolated from monocytes incubated with MPO-ANCA or control IgG ± LPS for 18 hours using a Qiagen RNeasy mini kit according to the manufacturer’s instructions. RNA (200 ng) was converted to cDNA using a RevertAid H Minus kit (Thermo Fisher Scientific) and stored at –80°C. Hydrolysis probes (TaqMan) were used (Thermo Fisher Scientific). The first strand cDNA obtained was amplified using the recommended TaqMan primer/probe mastermix in 384-well plates using the ABI Prism 7900 HT Sequence Detection System (Applied Biosystems) in duplicate. The sequence detector SDS 2.4 software (Applied Biosystems) was used to export the Ct values (threshold cycle). The reference gene bGus was used as an endogenous control. The comparative Ct method was used (2^–ΔΔCt^) for quantification.

### Statistics.

Prism (GraphPad software) was used for statistical analysis, with statistical tests used indicated in the figure legends. Some data were logarithmically transformed before analysis if the variances of the groups were significantly different. Differences were considered significant if *P* was less than 0.05. Where a Student’s *t* test was used it was 2 tailed.

### Study approval.

Blood samples from patients and healthy donors were taken following informed consent with ethical approval (NRES committee London—London Bridge 09/H084/72).

## Author contributions

RP, SH, MR, AT, and JW performed experiments. CS analyzed the mass spectrometry data. ML and AC generated and characterized the affinity-purified anti-MPO IgG. PL and BA analyzed the microarray data. MR, CK, and RP conceived the project and designed experiments. All authors analyzed data. MR wrote the manuscript. All authors edited the manuscript and approved the final version.

## Supplementary Material

Supplemental data

Supplemental table 2

## Version 1 01/26/2017

Electronic publication

## Version 2 08/22/2022

The volumes for the reported cell concentrations in Figure 6 were added. Figure legends 3, 5, and 7 were changed from mean to median fluorescence intensity
