# Supplementary material for: Anti-myeloperoxidase antibodies attenuate the monocyte response to LPS and shape macrophage development
Source: JCI Insight. 2017 Jan 26;2(2):e87379. doi: 10.1172/jci.insight.87379 (PMC5256146; doi:10.1172/jci.insight.87379)
Supplement: Supplemental data [file jciinsight-2-87379-s008.pdf]

Supplementary Table 1. Clinical characteristics of the patients donating the blood samples from which IgG was purified. The Birmingham Vasculitis Activity Score (BVAS) [1] for each patient is given, with the renal BVAS indicating they all had significant renal involvement. ENT Ear, nose and throat, NS nervous system. All samples were used for the experiment in Figure 1. The first 10 listed MPO-ANCA samples were used for the experiment in Figure 6A. The anti-MPO and anti-PR3 results are given from clinical data at the time of presentation. These results came from a range of different hospitals using different assays and so are not directly comparable. They do however given an indication as to whether the levels are low or high.

Age	Sex	ANCA	Anti-MPO or PR3 level (IU/ml)	Renal BVAS	Total BVAS	Extra renal disease
68	F	MPO	>100	12	22	ENT, Chest
72	F	MPO	188	12	18	Chest
55	F	MPO	115	12	15	No
77	F	MPO	>100	12	18	ENT, Chest
38	M	MPO	185	6	10	Eyes, Skin
66	M	MPO	67	12	18	Chest
82	F	MPO	144	12	15	No
74	F	MPO	294	12	12	No
73	F	MPO	166	12	12	No
74	F	MPO	19	12	15	No
63	M	MPO	>100	12	25	ENT, Eyes, Skin
42	M	PR3	211	12	35	ENT, NS, Eyes, Chest
74	M	PR3	231	12	31	ENT, NS, Skin, Chest
75	M	PR3	45	12	19	NS, Eyes, Skin
59	M	PR3	21	12	27	ENT, Eyes, Chest
76	M	PR3	581	12	19	Skin
51	M	PR3	84	12	23	Eyes, Chest
51	M	PR3	23	10	14	ENT, Eyes
73	M	PR3	31	12	19	Eyes, Skin
72	F	PR3	32	12	18	ENT

[1]Mukhtyar C, Lee R, Brown D, et al. Modification and validation of the Birmingham vasculitis activity score (version 3). Ann Rheum Dis 2009;68:1827-32.

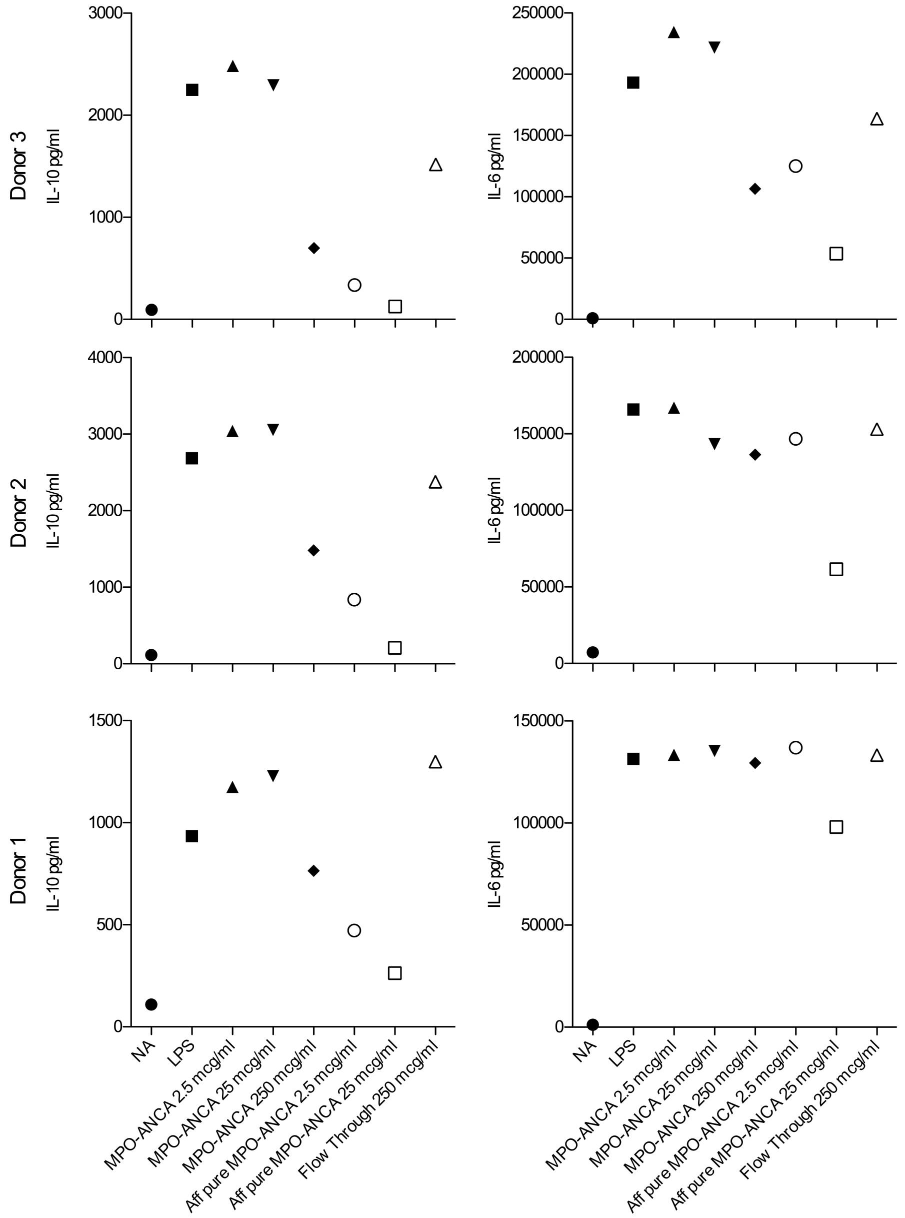

Supplementary Figure S1. IL-6 and IL-10 production from peripheral blood monocytes (3 donors) incubated for 18 hours with 100ng/ml LPS and MPO-ANCA, affinity purified (aff pure) MPO-ANCA, or flow through from the affinity purification. IgG (720mg) was purified from the plasma of a 56 year old female patient with BVAS = 25, MPO-ANCA = 126 IU/ml, using protein G. From this IgG (MPO-ANCA), approximately 1mg of affinity-purified MPO-ANCA was prepared using a Hitrap NHS HP column (GE Healthcare) loaded with human MPO (Biovitrum). ELISA plates (Nunc maxisorb) were coated with MPO and serial dilutions of the starting IgG preparation, affinity purified MPO-ANCA and flow through were applied. The ELISA data (not shown) demonstrated an approximately 300 fold increase in binding in the affinity purified MPO-ANCA compared to the starting MPO-ANCA. In all monocyte donors, MPO-ANCA reduced IL-10 at 250mcg/ml in keeping with our previous experiments, whereas a greater effect was seen with only 2.5mcg/ml of affinity purified MPO-ANCA. The effect on IL-6 was not consistent, with no effect for MPO-ANCA at 250 mcg/ml in donors 1 and 2. Despite this, a reduction in IL-6 was seen with only 25 mcg/ml of affinity purified MPO-ANCA in both cases. For donor 3 MPO-ANCA and affinity purified MPO-ANCA produced a reduction in IL-6 similar to that seen for IL-10. Overall these data were consistent with the enrichment seen on ELISA. As expected the flow through had less effect than the same concentration of MPO-ANCA in all cases. NA=not activated (no LPS), LPS= LPS only.

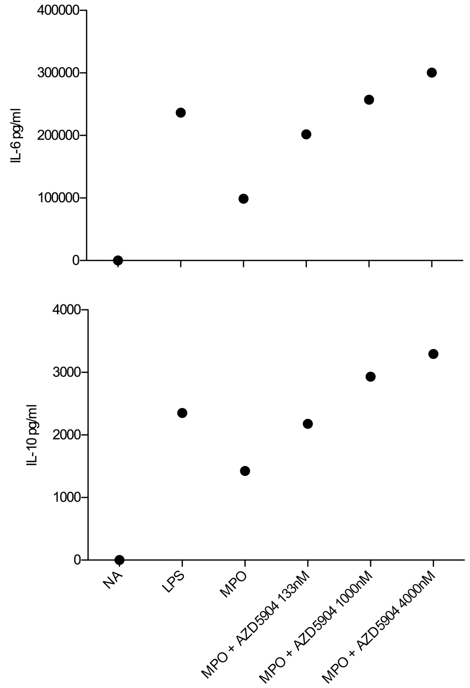

Supplementary Figure S2. Dose response curve for the effect of AZD5904 on IL-6 and IL-10 release from monocytes treated with LPS and exogenous MPO (2μg/ml) for 18 hours. The experiment was performed in the same way as that shown in Fig. 3A. NA=not activated (no LPS), LPS=LPS only.

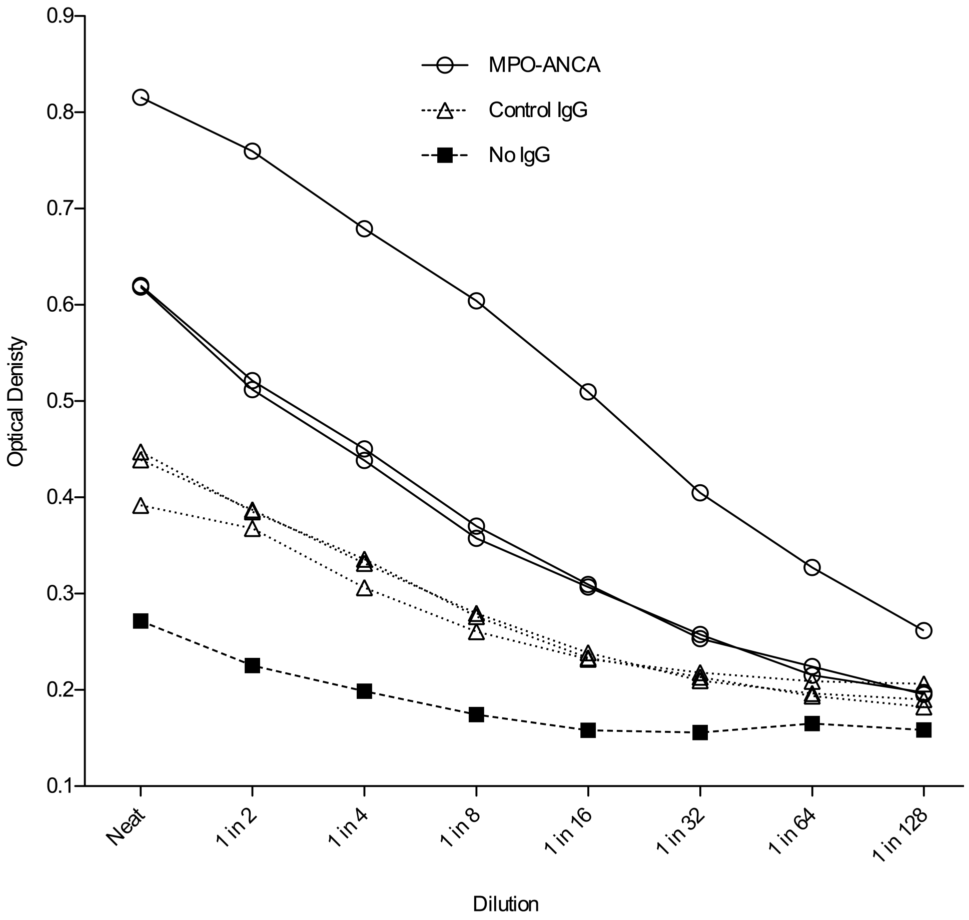

Supplementary Figure S3. Detection of immune complexes in the supernatant of monocytes activated with LPS in the presence of MPO-ANCA or control IgG (as in Fig. 1). The no IgG sample was from monocytes treated with LPS only. An ELISA was developed by combining two commercial ELISA kits. The coating antibody was from an MPO ELISA (R&D Systems, used as suggested by the manufacturer) and the detecting antibody was from an IgG ELISA (Bethyl laboratories, used at the dilution suggested by the manufacturer). Supernatants were applied to ELISA plates coated with anti-MPO at the indicated dilution and detected with anti-IgG. In this way we had an assay for MPO and IgG containing immune complexes. The data show that the readings were higher for the three samples containing MPO-ANCA compared to the three with control IgG. The latter gave higher readings than the supernatant with no IgG, which was probably due to background in the ELISA rather than immune complexes.

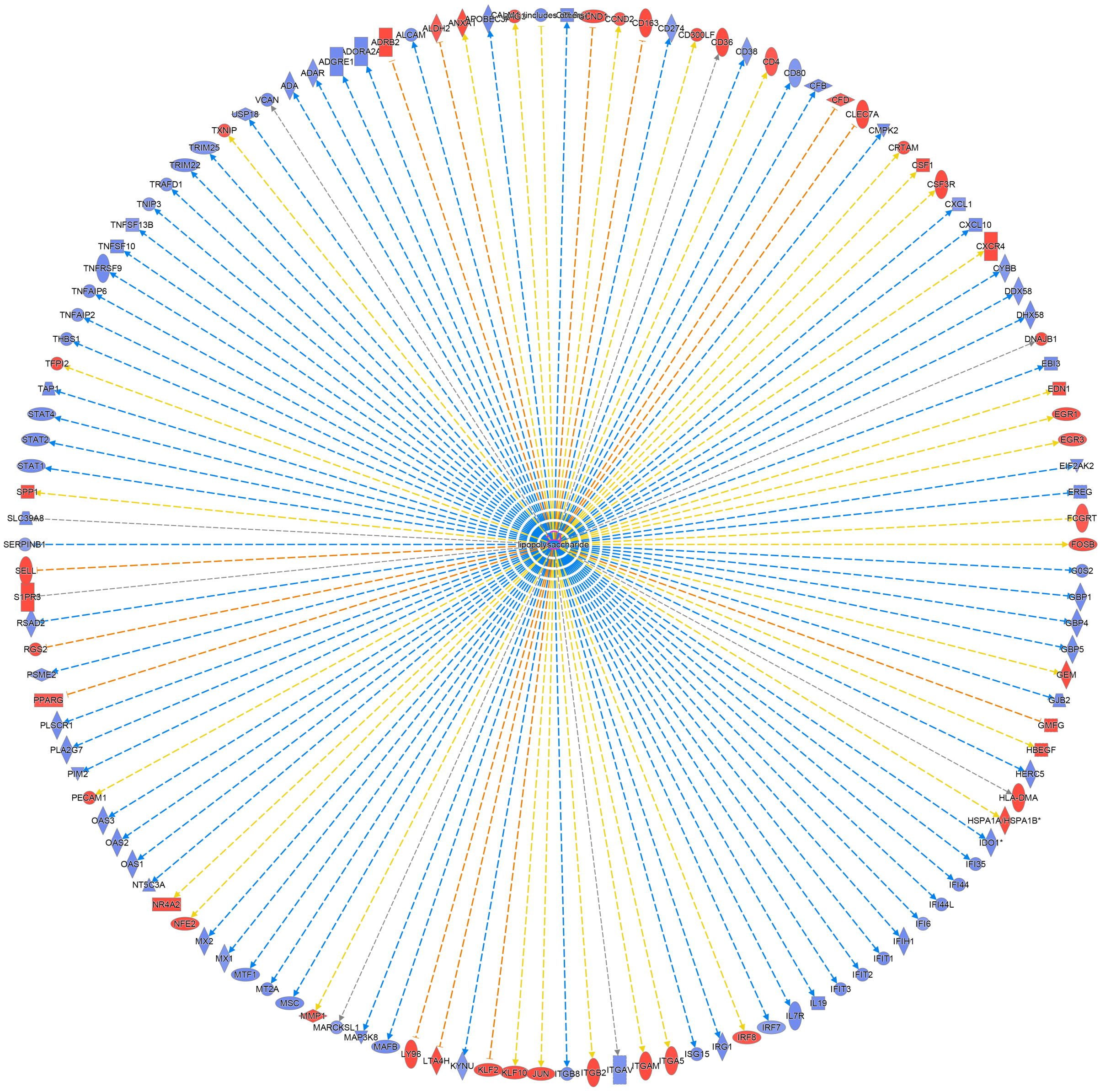

Supplementary Figure S4. Circle diagram of the effect of MPO-ANCA v Control IgG on genes in the LPS pathway, from the same experiment as that shown in Figure 4. For the depicted genes, blue = depressed by MPO-ANCA; red = enhanced by MPO-ANCA. The lines are predicted relationships where blue = depressed by LPS, and red is induced by LPS. Yellow is inconsistent with prediction; grey is not predicted.

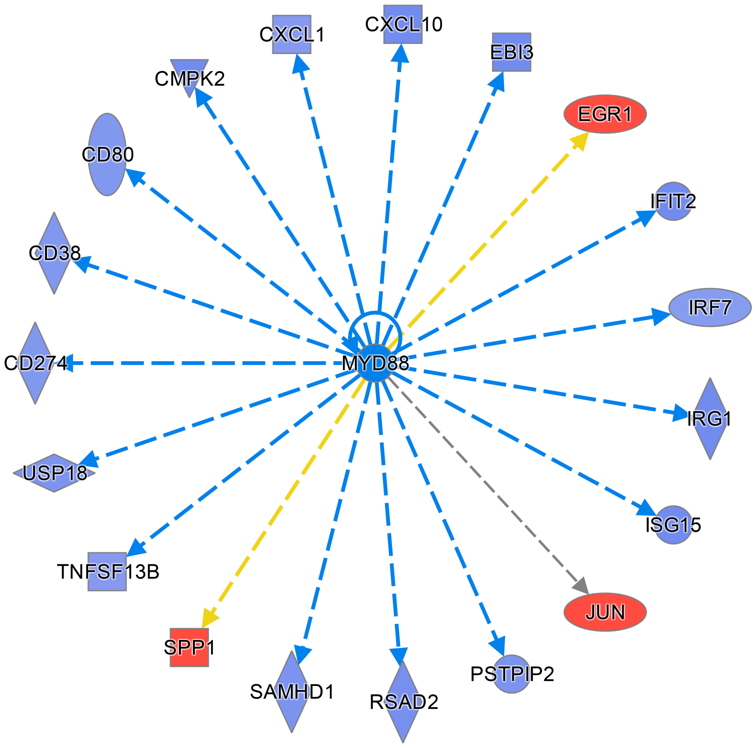

Supplementary Figure S5. Circle diagram of the effect of MPO-ANCA v Control IgG on genes in the Myd88 pathway, from the same experiment as that shown in Figure 4. For the depicted genes, blue = depressed by MPO-ANCA; red = enhanced by MPO-ANCA. The lines are predicted relationships where blue = depressed by Myd88, and red is induced by Myd88. Yellow is inconsistent with prediction; grey is not predicted.

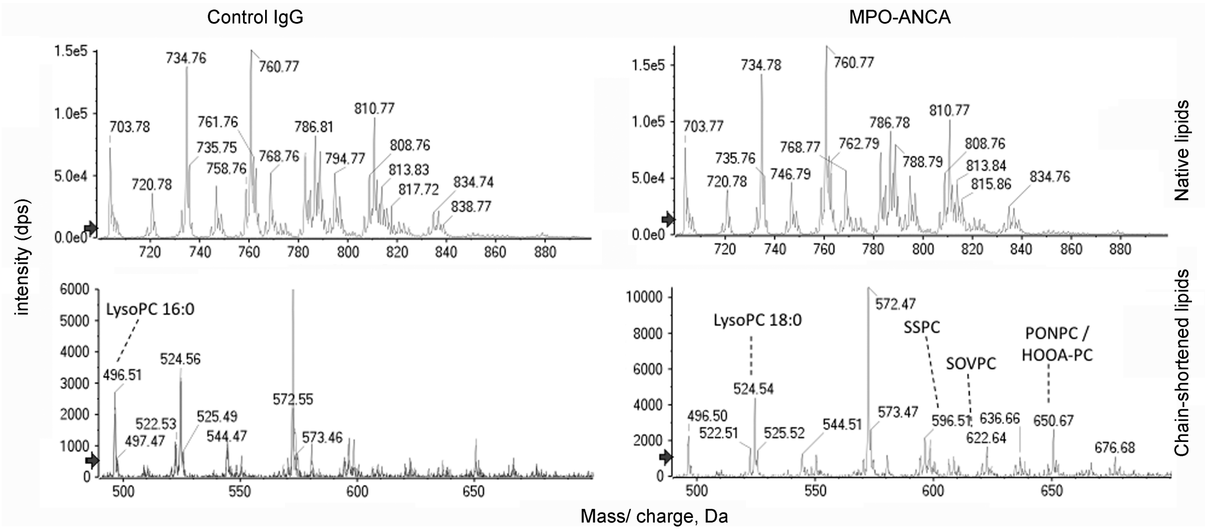

Supplementary Figure S6**.** Summed phospholipid spectra from treatments of monocytes with control IgG or MPO-ANCA (n=3 of each). Above are sum of spectra in the region m/z 700-900 corresponding to unmodified phospholipids from 3 control IgG samples and the 3 MPO-samples. Below are sum of spectra in the region m/z 490-700 corresponding to chain-shortened and oxidized phospholipids from 3 control IgG samples and the 3 MPO-ANCA samples. The XICs were prepared with a window of 0.7 Da and without smoothing. Abbreviations of lipid names are given in Supplementary Table 2.

Supplementary Table 3. Probable identities of native and oxidized phosphatidylcholine species detected in lipid extracts of monocytes by liquid chromatography mass spectrometry, based on retention time, m/z and previously published identifications. PC corresponds to sn-glycero-3-phosphocholine; plasmenyl corresponds to a vinyl ether linkage instead of an ester.

m/z	Chemical formula	Identification
**496.5**	$C_{24}H_{51}NO_7P^+$	Lyso-PC 16:0
**524.5**	$C_{26}H_{55}NO_7P^+$	Lyso PC 18:0
**544.5**	$C_{28}H_{51}NO_7P^+$	Lyso-PC 20:4
**572.5**	-	Unidentified
**596.5**	$C_{28}H_{55}NO_{10}P^+$	1-Palmitoyl-2-Succinoyl-PC (16:0, 4-COOH) *SSPC*
**622.5**	$C_{31}H_{61}NO_9P^+$	1-Stearoyl-2-Oxovaleroyl-PC (18:0, 5-CHO) *SOVPC*
**636.5**	$C_{32}H_{63}NO_9P^+$	1-palmitoyl-2-8-oxo-octanoyl PC (16:0, 8-CHO)
**650.5**	$C_{32}H_{61}NO_{10}P^+$	HOOA-PC (1-palmitoyl-2-5-hydroxy-8-oxooct-6-enoyl-PC)
	$C_{33}H_{65}NO_9P^+$	1-Palmitoyl-2-Oxononanoyl-PC (16:0, 9-CHO) *PONPC*
**666.5**	$C_{33}H_{65}O_{10}NP^+$	1-Palmitoyl-2-Azelaoyl-PC (16:0, 9-COOH) *PAzPC*
**720.8**	$C_{39}H_{79}NO_8P^+$	PC (31:0)
**720.8**	$C_{40}H_{83}NO_7P^+$	PC (16:0,16:0) ether
**734.8**	$C_{40}H_{81}NO_8P^+$	PC (16:0,16:0) 1,2-Dialmitoyl-PC *DPPC*
**746.8**	$C_{42}H_{85}NO_7P^+$	PC (16:0, 18:1) Plasmenyl
**758.8**	$C_{42}H_{81}NO_8P^+$	PC (16:0/18:2)
**760.8**	$C_{42}H_{83}NO_8P^+$	PC (16:0,18:1)
**762.8**	$C_{42}H_{85}NO_8P^+$	PC (16:0, 18:0)
**768.8**	$C_{43}H_{79}NO_8P^+$	PC (16:0. 20:4) Plasmenyl
**782.8**	$C_{44}H_{81}NO_8P^+$	PC (16:0/20:4) *PAPC*
**786.8**	$C_{44}H_{85}NO_8P^+$	PC (18:0/18:2)
**788.8**	$C_{44}H_{87}NO_8P^+$	PC (18:0/18:1)
**798.8**	$C_{44}H_{81}NO_9P^+$	PC (16:0/20:4)-OH
**806.8**	$C_{46}H_{81}NO_8P^+$	PC (16:0/22:6)
**810.8**	$C_{46}H_{85}NO_8P^+$	PC (18:0/20:4)
**828.8**	$C_{44}H_{79}NO_{11}P^+$	1-palmitoyl-2-(5,6-epoxyisoprostane E_2)-PC *PEIPC*
**834.8**	$C_{48}H_{85}NO_8P^+$	PC (18:0/22:6)

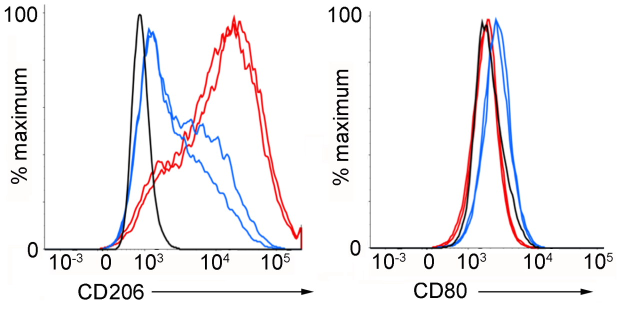

Supplementary Figure S7. Expression of CD206 and CD80 on monocytes/macrophages developed after 3 days in culture with 10% AB serum with control IgG (n=2), or MPO-ANCA (n=2). Experiments were performed with monocytes from 2 healthy donors with histograms shown for one donor. CD206 Median fluorescence intensities (MFIs) for donor 1 were 7696, 9974 (MPO-ANCA) and 3597, 2529 (Control IgG). CD206 MFIs for donor 2 were 11773, 14449 (MPO-ANCA) and 1953, 2558 (Control IgG). CD80 MFIs for donor 1 were 1801, 1699 (MPO-ANCA) and 2391, 2505 (Control IgG). CD80 MFIs for donor 2 were 1939, 1928 (MPO-ANCA) and 1725, 2107 (Control IgG). The CD206 results were therefore consistent with day 6 results in both donors, with the CD80 results more variable. Histograms for donor 1 are shown.

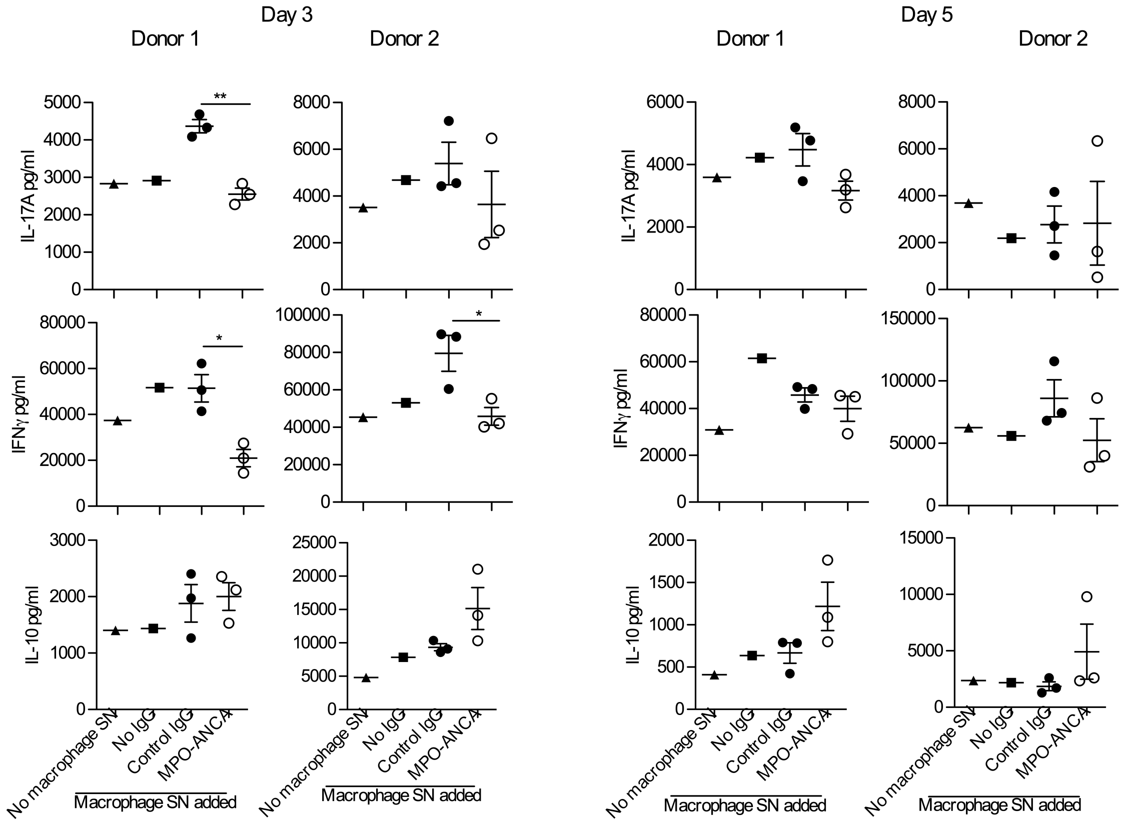

Supplementary Figure S8. Peripheral blood CD4+ T cells were stimulated for 3 and 5 days with anti-CD3 and anti-CD28 in the presence or absence of supernatants of macrophages that developed from monocytes after 6 day in culture with 10% AB serum and MPO-ANCA or control IgG. The ratio of IL-10 to IFNγ for these experiments are in figure 7 in the main paper, with raw data giving concentrations of IL-17A, IFNγ and IL-10 shown here. *p<0.05, **p<0.01, **p<0.001 (two-tailed student's t test). Error bars represent mean ± SEM.
